# Crystallographic contributions to piezoelectric properties in PZT thin films

**DOI:** 10.1038/s41598-019-43869-1

**Published:** 2019-05-13

**Authors:** Goon Tan, Kazuki Maruyama, Yuya Kanamitsu, Shintaro Nishioka, Tomoatsu Ozaki, Toshihito Umegaki, Hirotaka Hida, Isaku Kanno

**Affiliations:** 1 Kobe University, 1-1 Rokkodai-cho, Nada-ku, Kobe, 657-8501 Japan; 2 Technology Research Institute of Osaka Prefecture, 2-7-1 Ayumino, Izumi city, Osaka 594-1157 Japan

**Keywords:** Actuators, Electronic devices

## Abstract

We report on the correlated investigation between macroscopic piezoelectric properties and the microscopic deformation of crystal structures of both epitaxial and polycrystalline Pb(Zr,Ti)O_3_ (PZT) thin films grown on MgO and Si substrates, respectively. We observed the reversible elongation and contraction of lattice parameter under an applied electric field using synchrotron X-ray diffraction. The effective piezoelectric coefficients were estimated from the relationship between electric field and field-induced strain, and compared with those characterized by the macroscopic cantilever method. The electric field dependences of the piezoelectric coefficients obtained from both characterization were in good agreement with each other. The results also revealed large and nonlinear piezoelectric properties for the polycrystalline PZT thin film. The comparative discussion in this study provides valuable insights of crystallographic contributions and opens the way to improve the piezoelectricity in thin-film based piezoelectric devices.

## Introduction

Piezoelectric materials, such as lead zirconate titanate Pb(Zr,Ti)O_3_ (PZT) for example, provide the means to convert between mechanical and electrical energies that arise from direct and converse piezoelectric effects. For PZT, the composition in the morphotropic phase boundary (MPB), which is at the interface between tetragonal (*P4mm*) and rhombohedral (*R3m*) phases, provides superior dielectric and piezoelectric properties for a variety of technological applications^1,2^. PZT thin films have been widely investigated for their use in microelectromechanical systems (MEMS) such as sensors, actuators, and energy harvesters^3–10^. It is well known that the piezoelectric properties of PZT thin films are different from those of their bulk ceramics counterparts because their characteristics are affected by other factors, including film thickness, crystal orientation, and substrate dependent internal stresses^11–13^. Therefore, an investigation into the fundamental properties of PZT piezoelectric thin films is of great significance to improve MEMS devices.

Much efforts have focused on the origin of piezoelectric effect via *in-situ* characterization methods. Recently, we have developed a simple and precise method to measure the effective piezoelectric coefficients (*e*_31,*f*_) of PZT thin films using unimorph cantilevers^14–17^. The piezoelectric properties consist of intrinsic and extrinsic contributions^7^. The intrinsic contribution is concerned with crystal lattice deformation (such as elongation and shrinkage), while the extrinsic contribution originates from collective lattice distortions caused by domain rotations or phase transitions. Our previous report demonstrated that the piezoelectric properties of polycrystalline PZT thin films was larger than that of epitaxial PZT thin films^16^. In this study, to understand the crystallographic factors contributing to the macroscopic piezoelectric properties, we performed *in-situ* X-ray diffraction (XRD) measurements^18–24^ on PZT thin films using a synchrotron radiation source. Here, we demonstrate one of the first comparative discussions that evaluates the macroscopic piezoelectric properties and the microscopic deformation of crystal structures of epitaxial and polycrystalline Pb(Zr,Ti)O_3_ (PZT) thin films. Our study quantitatively clarifies the crystallographic contributions needed to enhance piezoelectricity in thin-film based devices.

## Results and Discussion

We prepared epitaxial PZT thin films on (001)Pt/MgO substrates and polycrystalline PZT thin films on (111)Pt/Ti/SiO_2_/Si substrates. Both films were approximately 3 μm thick and were fabricated using rf-magnetron sputtering. The Zr/Ti ratio was approximately 52/48, which is near the MPB composition. The crystal structures were confirmed using conventional XRD analysis methods (Supplementary Section 1). The epitaxial and polycrystalline PZT thin films were both found at a *c*-axis orientation. For the PZT thin film grown on the MgO substrate, we could only observe the tetragonal spot in the reciprocal lattice map (Supplementary Fig. S1.1(b)). This result represents that the crystal phase was mainly tetragonal because of the large in-plane stress from the MgO substrate^12^.

**Figure 1:**
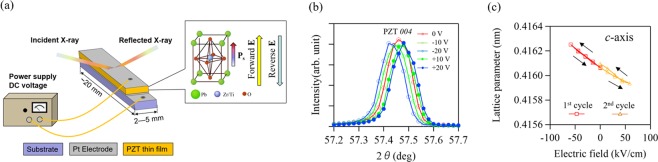
(**a**) A schematic illustration of the experimental setup for out-of-plane XRD measurements under DC voltages. The arrows next to the crystal structure depict the direct relationships between polarization and applied electric field. (**b**) Out-of-plane XRD patterns of the epitaxial PZT thin film obtained at the PZT *004* peak under varying DC voltages. (**c**) Variation in *c*-axis lattice parameter as a function of electric field.

*In-situ* XRD measurements were carried out using synchrotron radiation (λ = 0.1 nm) performed at beamlines BL19B2 and BL46XU in the SPring-8 synchrotron research facility, Japan. Figure 1(a) shows a schematic illustration of the experimental setup for *in-situ* XRD measurements in the surface normal (out-of-plane) direction. Here, specimens were not subjected to electrical poling treatments before a measurement was taken. Note that the initial polarization direction of the as-deposited PZT thin film was in the thickness direction^25^, as depicted in Fig. 1(a). Hence, when a negative voltage was applied to the top Pt electrode, it corresponds to forward bias. First, we applied negative voltages (forward bias) up to −20 V for the 1^st^ cycle, and then applied positive voltages (reverse bias) up to +20 V for the 2^nd^ cycle. Figure 1(b,c) show the PZT *004* peak under varying DC voltages, and the variation in *c*-axis lattice parameter determined by the observed peak positions. As can be seen in the XRD pattern, the diffraction peaks shift toward lower angles with increasing negative voltage, and vice versa. In order to confirm the shifts were not resulted from the movement of the film surface, we also observed the XRD pattern for Pt electrode layer. We ascertained that the diffraction peak of Pt electrode remained unchanged under the applied voltage (Supplementary Fig. S2(a)). Therefore, the observed PZT peak shifts were caused only by the deformation of the PZT crystal lattice due to the converse piezoelectric effect. The relative changes in the *c*-axis lattice parameter as a function of electric field represents the reversible elongation and contraction of the crystal structure along the electric field bias magnitude and direction. We also observed a small PZT *400* tetragonal peak in the XRD pattern, which confirms the constancy in the peak position and peak area ratios between *004* and *400* peaks (Supplementary Fig. S3(b,c)). This indicates an absence of a 90° domain rotation in the epitaxial PZT thin film. Therefore, the epitaxial PZT thin film was considered firmly clamped due to the large compressive stress from the MgO substrate^12^. An absence in domain rotation is also consistent with a previous study on epitaxial PZT thin films grown on CaF_2_ substrates^18^.

**Figure 2:**
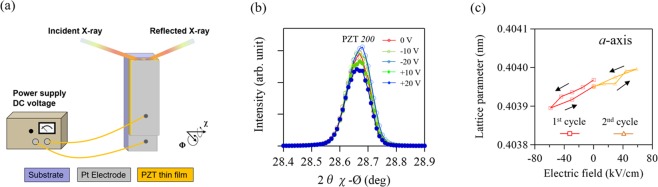
(**a**) A schematic illustration of the experimental setup for in-plane XRD measurements under varied DC voltages. (**b**) In-plane XRD patterns obtained at the PZT *200* peak under varying DC voltages. (**c**) Variation in *a*-axis lattice parameter as a function of electric field.

**Figure 3:**
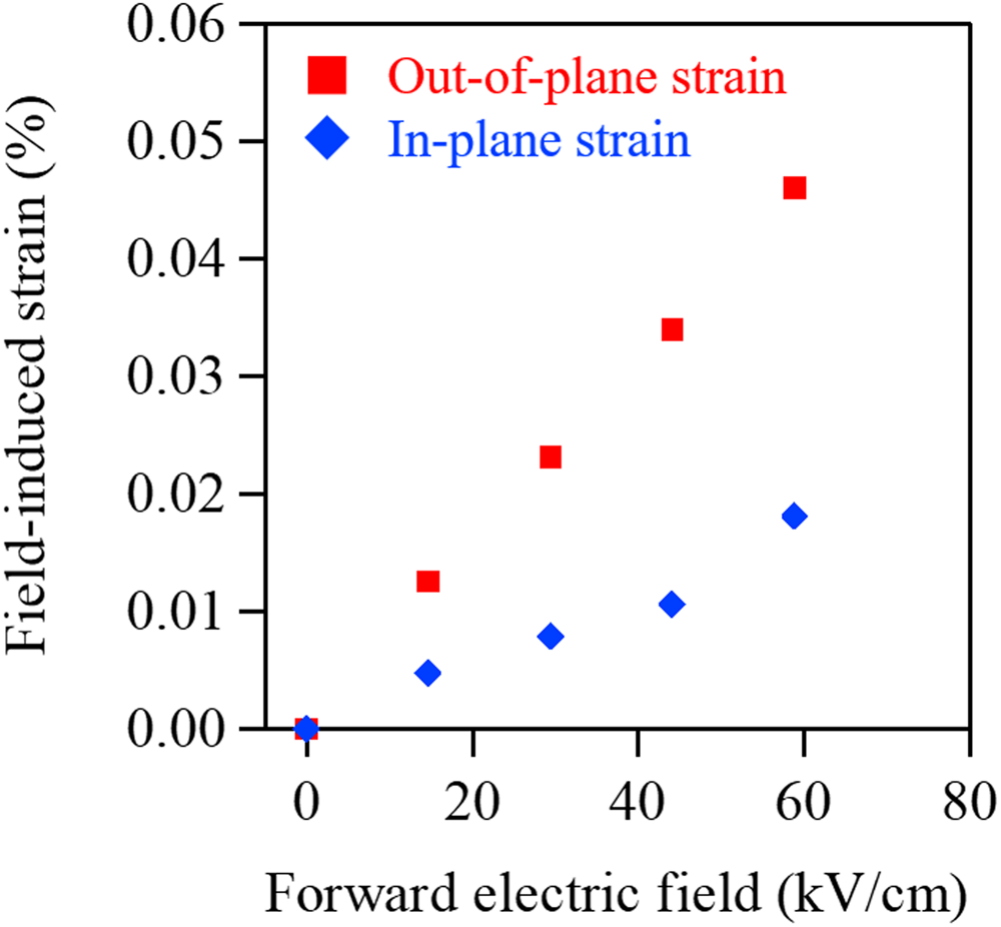
Field-induced strain as a function of forward electric field.

For in-plane measurements, the specimen was adjusted to the vertical position by χ-axial rotation, as illustrated in Fig. 2(a). We confirmed the synchrotron X-ray beam irradiated the entire PZT thin film as an MgO peak was observed to align in the direction of irradiation. Figure 2(b,c) display an XRD pattern of the in-plane PZT *200* peak at various applied voltages, and the changes observed in the in-plane *a*-axis lattice parameter. We ascertained the reversible contraction and elongation behavior of the *a*-axis lattice parameter. The electric field dependence of the in-plane lattice parameter indicates the behavior opposite to that of out-of-plane case, as shown in Fig. 1(c). This result is consistent with the fact that when the out-of-plane crystal lattice is stretched along the forward electric field, the in-plane crystal lattice shrinks, and vice versa. Figure 3 shows the field-induced lattice strain calculated from the lattice variations. Both out-of-plane and in-plane strains exhibit a good linear dependence on the electric fields, suggesting an effect on the piezoelectric responses. The average strain ratio between the out-of-plane and in-plane lattice (Fig. 3) was approximately 0.36. This is similar to that of the literature value for Poisson’s ratio (*v*_*s*_ = 0.39) in PZT thin films^26^.

**Figure 4:**
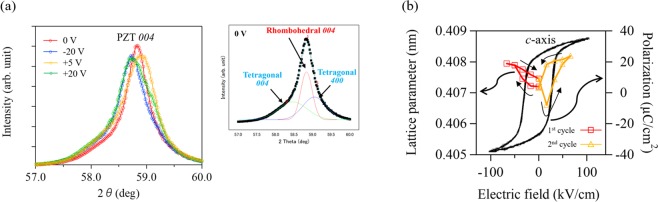
(**a**) Out-of-plane XRD patterns of the polycrystalline PZT thin film at the PZT *004* peak under varying DC voltages. The inset shows fitted curves of PZT peaks of rhombohedral and tetragonal phases. (**b**) The variation in *c*-axis lattice parameter as a function of electric field, superimposed with the *P*-*E* hysteresis curve measured at 1 kHz.

**Figure 5:**
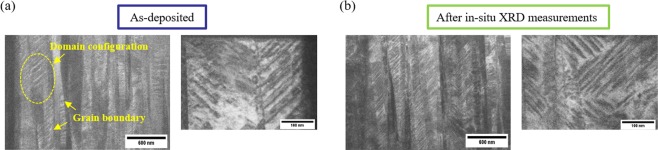
Dark field STEM images of (**a**) the as-deposited PZT thin film and (**b**) the thin film following *in-situ* XRD measurements after applied voltages cycles of up to ±30 V. The insets in (**a**,**b**) show the enlarged views.

Then, we investigated the polycrystalline PZT thin film on a Si substrate. The experimental setup was similar to that of the epitaxial PZT thin films. We also applied negative voltages (forward bias) of up to −20 V for the 1^st^ cycle, and positive voltages (reverse bias) of up to +20 V for the 2^nd^ cycle. We confirmed the position of the Pt *111* peak remained unchanged under the applied voltages (Supplementary Fig. S2(b)). Figure 4(a) displays a synchrotron XRD *θ–*2*θ* pattern around the PZT *004* peak under varied DC voltages. As can be seen in Fig. 4(a), the polycrystalline PZT *004* peak is much broader in comparison to the epitaxial PZT peak (Fig. 1(b)). The broad peak also indicates the existence of a slight tetragonal *P4mm 400* and *004* phase, as shown in the inset. Figure 4(a) reveals a clear shift in the diffraction peak for both positive and negative biases, and the peaks are shifted toward a higher angle only when applying a +5 V. Figure 4(b) shows the field-induced variation of the *c*-axis lattice parameter superimposed with the polarization-electric field (*P*-*E*) hysteresis loop measured at 1 kHz. The *c*-axis lattice parameter exhibits a butterfly curve that arises from the inverse polarization effect. This behavior is consistent with the coercive electric field determined from the *P*-*E* hysteresis loops. It should be noted that the coercive electric field of the polycrystalline PZT thin film is much smaller than that of the epitaxial PZT thin film. We also applied further forward and reverse biases for the 3^rd^ and 4^th^ cycles up to ±30 V and determined similar elongation and contraction behavior for the *c*-axis lattice parameter (Supplementary Fig. S4). Following the *in-situ* XRD measurements, we analyzed the domain structures and compared them to the as-deposited polycrystalline PZT thin film using scanning transmission electron microscopy (STEM). Dark field STEM images of the specimen cross-sections were taken at the *001* reflection. Figure 5(a) displays columnar shaped grains and local domain configurations contained within the as-deposited film. Conversely in Fig. 5(b), the domain density was found to increase after 4 different voltage cycles (up to ±30 V) in the *in-situ* XRD measurements. An increase in domain density suggests that significant changes to the domain orientation or phase transition occurred through the applied voltages. The variation in domain structures indicates enhanced mobility of polarization direction for the polycrystalline PZT thin films at MPB composition. We also carried out in-plane XRD measurements for the polycrystalline PZT thin films. Unfortunately, we were unable to observe an effective peak shifts, possibly due to the random in-plane orientations inherent in polycrystalline films (Supplementary Fig. S1.2(b)).

**Figure 6:**
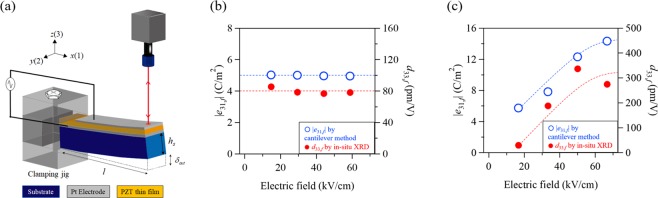
(**a**) Schematic illustration of the setup for determining macroscopic piezoelectric coefficients (*e*_31,*f*_) using the cantilever method. (**b**) Comparison of piezoelectric coefficients for the epitaxial PZT thin film. (**c**) Comparison of piezoelectric coefficients for the polycrystalline PZT thin film.

Next, we compared the piezoelectric coefficients obtained from the *in-situ* XRD measurements with those evaluated by the cantilever method. The effective piezoelectric coefficients (*d*_33,*f*_) for the epitaxial and polycrystalline PZT thin films were estimated from the relationship between electric field and field-induced strain. We used the formula ${\rm{\Delta }}x={d}_{33,f}\times E$, where Δ*x* is the unit cell displacement that corresponds to the induced strain. The macroscopic piezoelectric coefficients (*e*_31,*f*_) of the PZT thin films were measured using the cantilever method as illustrated in Fig. 6(a). From the input voltage, *V*_*in*_ and the output displacement in the 3 (z-axis) direction, *δ*_*out*_, we calculated *e*_31,*f*_ using Eq. (1) as follows:

1 $$\,{e}_{31,f}=-\,\frac{{E}_{s}{h}_{s}^{2}}{3{l}^{2}(1-{\nu }_{s})}\cdot \frac{{\delta }_{out}}{{V}_{in}},$$

where *E*_*S*_, *h*_*S*_ and *v*_*s*_ are the Young’s modulus, thickness, and Poisson’s ratio of the base substrate, respectively. Here, *l* denotes the length of the cantilever. The derivation of Eq. (1) is described in detail in our previous paper^16^. Figure 6(b) compares the piezoelectric coefficients for the epitaxial PZT thin film. In this case, the electric field dependency of |*e*_31,*f*_| was almost constant; at approximately 5.0 C/m^2^. We confirmed that the *d*_33,*f*_ estimated from *in-situ* XRD measurements was also constant. The average *d*_33,*f*_ value was found to be approximately 80 pm/V, which was similar to those of previous studies^18,19^. On the other hand, the piezoelectric coefficients of the polycrystalline PZT thin films placed a significant dependence on the applied electric field (Fig. 6(c)). The |*e*_31,*f*_| increased proportionally with the electric field and gradually plateaued at higher electric fields. This behavior is consistent with our previous study^16^. The |*e*_31,*f*_| value ranged from 6–14 C/m^2^, which was higher than that of the epitaxial PZT thin film. The estimated *d*_33,*f*_ also presented a similar reliance on the electric field, as shown in Fig. 6(c). The *d*_33,*f*_ value here ranged from 30–340 pm/V. This high, non-linear behavior was attributed to large extrinsic effects, such as field-induced phase transitions^23,27,28^ and incremental domain densities^29^. Our study demonstrates an increase in domain density via applied electric fields (Fig. 5(b)). This result suggests that the easy rotation of the polarization direction at the MPB region for polycrystalline PZT thin films plays an important role to induce high piezoelectric properties. The polarization rotation accompanied by a crystalline phase transition and domain reorientation has the potential to strongly enhance the macroscopic piezoelectric properties.

In summary, our study verifies the value of direct observations for crystallographic deformation through *in-situ* XRD measurements. Comparative discussions were also obtained with macroscopic piezoelectric coefficients using the cantilever method. The electric field dependences of the piezoelectric coefficients attained from both *in-situ* XRD results and the cantilever method agree well each other. Both evaluations indicate that the piezoelectric property of the polycrystalline PZT thin film was larger than that of the epitaxial PZT thin film. We infer that the enhanced macroscopic piezoelectric property of the polycrystalline PZT thin film is attributed to the easy rotation of the polarization direction, accompanied by a crystal phase transition and domain reorientation. This study highlights the importance of comparative discussions to improve the piezoelectric properties of piezoelectric thin films for the application of novel piezoelectric MEMS devices.

## Methods

Pb(Zr,Ti)O_3_ (PZT) thin films with a Zr/Ti ratio of approximately 52/48 were prepared on (001)Pt/MgO and (111)Pt/Ti/SiO_2_/Si substrates using the rf-magnetron sputtering system. To stabilize the PZT growth, 50 nm-seed layers of 10%-La doped PbTiO_3_ and SrRuO_3_ were deposited for Pt/MgO and Pt/Ti/SiO_2_/Si, respectively, before the deposition of the PZT thin films. The growth conditions of the PZT thin films were as follows: The substrate temperature was held at about 550 °C while the chamber pressure was kept at 0.3 Pa in a mixed gases of Ar/O_2_ with the flow ratio of 39/1 sccm. The distance between substrate and target was 5 cm. The deposition rate was approximately 600 nm/h and resulting thickness was about 3 μm, measured by a stylus profiler (Dektak-8, Veeco). Before *in-situ* XRD measurements, the crystalline structures of the PZT thin films were characterized using a conventional XRD (Cu*Kα* radiation source, Rigaku).

The transverse piezoelectric coefficients (*e*_31,*f*_) of epitaxial and polycrystalline PZT thin films were measured using the cantilever method. After depositing large-area Pt top electrodes on the film surfaces, the substrates were diced into rectangular shapes. Then, we clamped one end of each specimen onto a vise and connected the top and bottom of the Pt electrodes to a power source. Next, we applied negative unipolar sinusoidal voltages from 5 V to 20 V to the top electrode and measured the tip displacement of the cantilever through a Laser Doppler Vibrometer. Then, we measured the converse piezoelectric properties of the thin films at a frequency of 290 Hz, which was sufficiently lower than the resonance frequency. The ferroelectricity of the PZT thin films was confirmed by Sawyer-Tower circuit tests.

The *in-situ* XRD experiments were undertaken using synchrotron radiation with a 12.4 keV photon energy (λ = 0.1 nm) on the BL19B2 and BL46XU beamlines at the SPring-8 synchrotron research facility, Japan. DC voltages were applied during the *in-situ* XRD measurements and Au wires were bonded to the top and bottom of the Pt electrode layer. A DC power source was connected to the Au wires, which provided a constant DC electric field during the *in-situ* XRD measurement. Specimens were firmly fixed onto the stage with double sided tape. All the measurements were carried out at room temperature.

The samples prepared for STEM analysis were done so using Focused ion beam milling (Hitachi FB-2200). Then, domain observations were carried out using a spherical aberration corrected STEM (Hitachi HD-2700) microscope, operated at 200 kV.

## Supplementary information

Crystallographic contributions to piezoelectric properties in PZT thin films
