# Supplementary material for: Crystallographic contributions to piezoelectric properties in PZT thin films
Source: Sci Rep. 2019 May 13;9:7309. doi: 10.1038/s41598-019-43869-1 (PMC6514214; doi:10.1038/s41598-019-43869-1)
Supplement: Supplementary file 1 — Crystallographic contributions to piezoelectric properties in PZT thin films [file 41598_2019_43869_MOESM1_ESM.docx]

**Supplementary information**

**Crystallographic contributions to piezoelectric properties in PZT thin films**

Goon Tan^1^, Kazuki Maruyama^1^, Yuya Kanamitsu^1^, Shintaro Nishioka^1^, Tomoatsu Ozaki^2^, Toshihito Umegaki^1^, Hirotaka Hida^1^ & Isaku Kanno^1^*

^1^Kobe University, 1-1 Rokkodai-cho, Nada-ku, Kobe 657-8501, Japan

^2^Technology Research Institute of Osaka Prefecture, 2-7-1 Ayumino, Izumi city, Osaka 594-1157, Japan

* To whom correspondence should be addressed

Email: kanno@mech.kobe-u.ac.jp

**1. Preliminary X-ray diffraction (XRD) studies before in-situ synchrotron XRD measurements**

**1.1 PZT thin film grown on the MgO substrate**

A wide angle *θ*–2*θ* scan of the Pb(Zr,Ti)O_3_ (PZT) thin film grown on the (001)Pt/MgO substrate revealed that the thin film structure formed a single tetragonal phase in the (001)-orientation with the absence of the pyrochlore phase, as shown in Fig. S1.1(a). Reciprocal space mapping (RSM) showed clear PZT spots around *004* and *024* reciprocal lattice diffractions (Fig. S1.1(b)), verifying the presence of epitaxial growth. The RSM around the *024* reciprocal lattice diffraction indicates that both the bottom Pt electrode layer and the PZT thin film are fully relaxed on the MgO substrate. We note that the film thickness of PZT was approximately 3 μm.

**
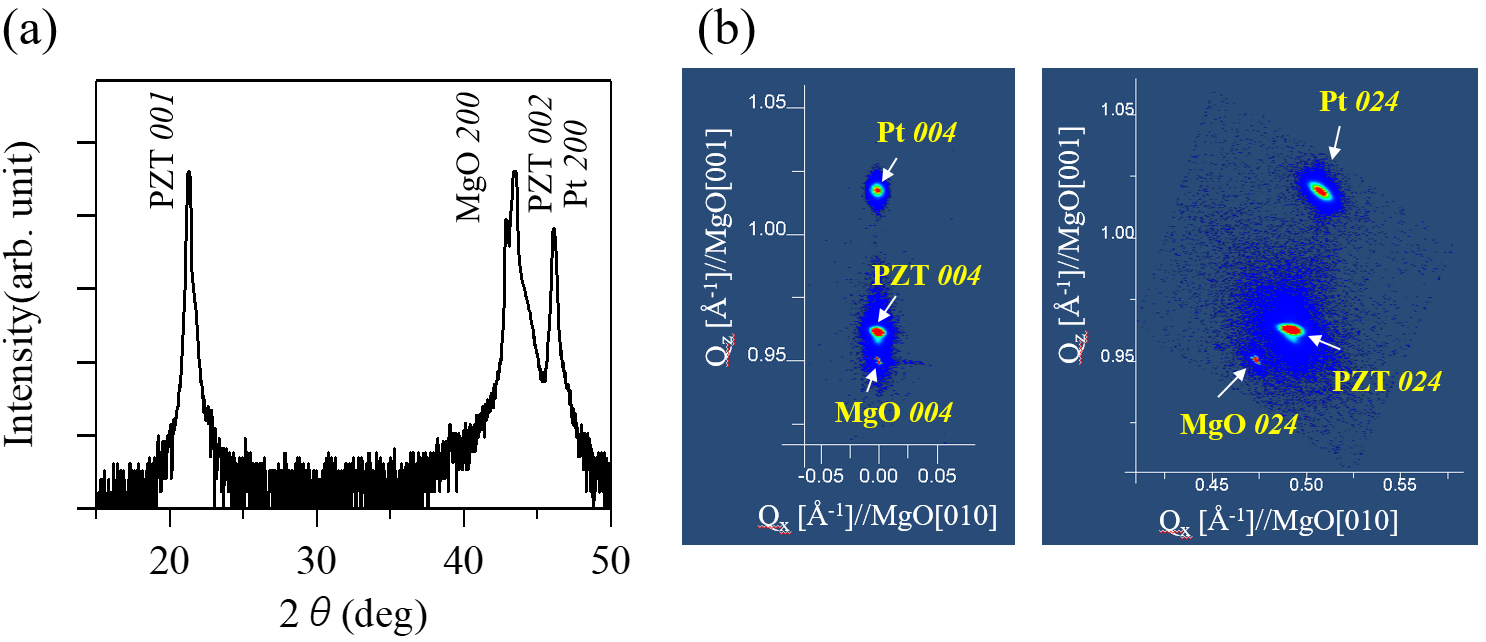
**

**Figure S1.1.** (a) Wide angle *θ*–2*θ* scan of the PZT thin film on the (001)Pt/MgO exhibits a (001)-oriented single tetragonal phase. (b) Reciprocal space maps of the PZT thin film on (001)Pt/MgO around the *004* and *024* spots. The RSM around the *024* reciprocal lattice diffraction indicates both the bottom Pt electrode layer and the PZT thin film are fully relaxed on the MgO substrate.

**1.2 PZT thin film grown on the Si substrate**

A wide angle *θ*–2*θ* scan of the PZT thin film on the (111)Pt/Ti/SiO_2_/Si substrate revealed a polycrystalline perovskite structure with a *c*-axis preferred orientation (Fig. S1.2(a)). Figure S1.2(b) showed random orientation at the in-plane 2*θχ*/*Φ* scan.

**
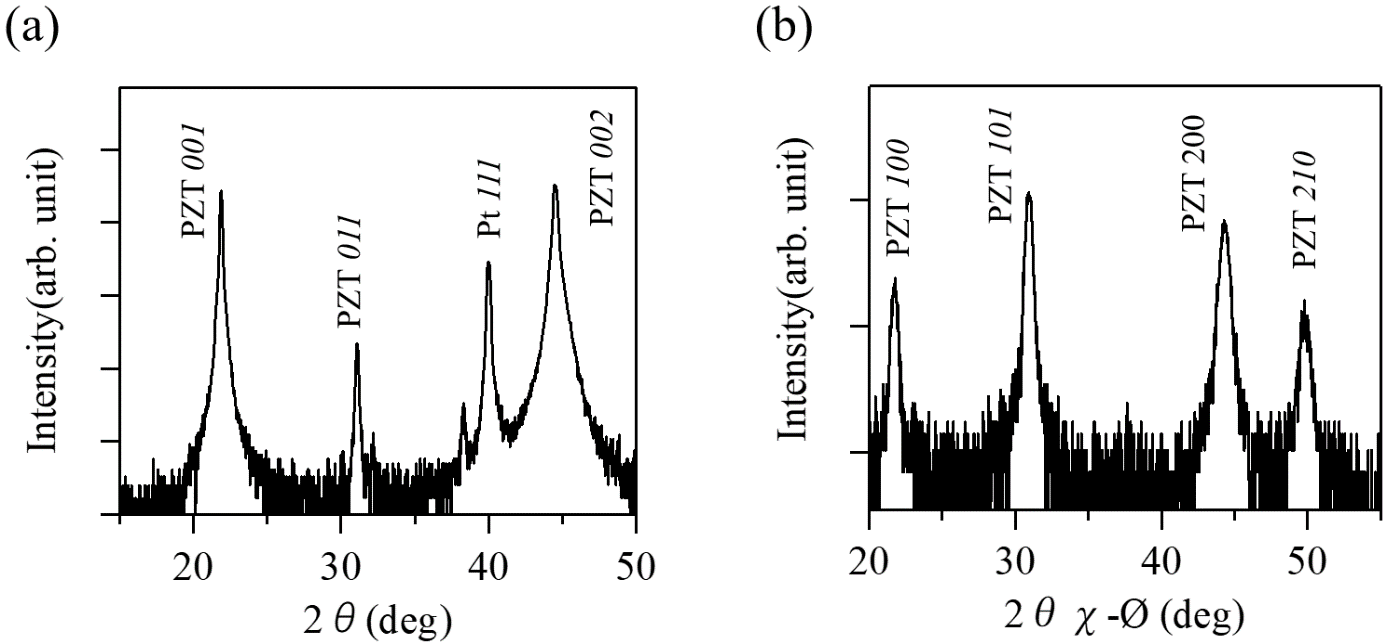
**

**Figure S1.2.** (a) Wide angle *θ*–2*θ* scan of the PZT thin film on the (111)Pt/Ti/SiO_2_/Si revealing a polycrystalline structure with *c*-axis preferred orientation. (b) An in-plane 2*θχ*/*Φ* scan shows random in-plane crystal orientation.

**2. In-situ XRD observations of Pt peaks**

Similar to the results of the in-situ XRD measurements, we also analyzed the Pt electrode XRD patterns for both polycrystalline and epitaxial PZT thin films. Here, the Pt electrode layer grown on the MgO substrate exhibits a (001)-orientation, whilst the Pt layer grown on the Si substrate exhibits a (111)-orientation. We confirm that no shifts were observed for both Pt peaks, and the calculated lattice parameters remained constant as a function of applied voltage (Fig. S2). This indicates the PZT peak shifts as shown in the main text arose from the deformation of the PZT crystal structure via the converse piezoelectric effect.

**
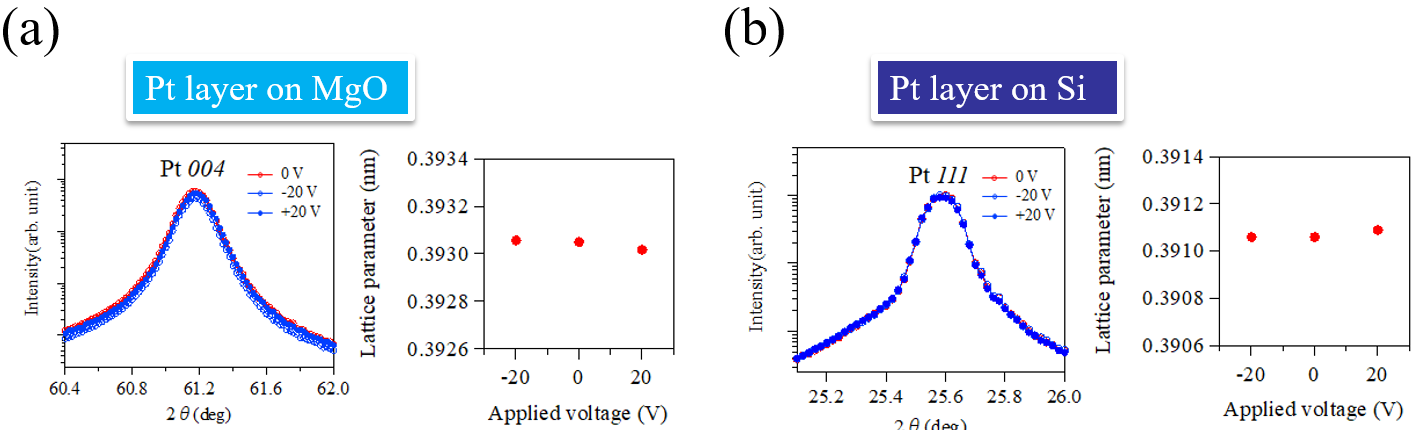
**

**Figure S2.** In-situ XRD scans of (a) the Pt *004* peak on the MgO substrate and (b) the Pt *111* peak on the Si substrate. We confirm that the Pt peaks did not shift in both cases under the applied voltages. The calculated lattice parameters remained constant against the applied voltages. This indicates the peak shifts in PZT thin films arose from the deformation of the PZT crystal structure via the converse piezoelectric effect.

**3. In-situ XRD measurements around the PZT *400* peak of the epitaxial PZT thin film**

Figure S3 shows the out-of-plane XRD measurements around the PZT *400* tetragonal peak with varied voltages applied. Unlike the clear *004* peak shift shown in the insert, we establish that the *400* peak position and the calculated lattice parameter remained unchanged, as seen in Fig. S3(a) and (b). Then, we evaluated the *c*-domain volume fraction (*V_004_*) from the area ratio between the *400* and *004* peaks of PZT. The *V_004_* (%) was estimated using the formula [$V_{004}=A(004)\times100/\{A(004)+A(400)\}$]. Here, *A* denotes the peak area. As shown in Fig. S3(c), the initial *V_004_* was approximately 99.7%, which was almost constant for both positive and negative electric fields. This result indicates that most of the domains were *c*-oriented and the 90˚ domain rotation was suppressed in the epitaxial PZT thin film.


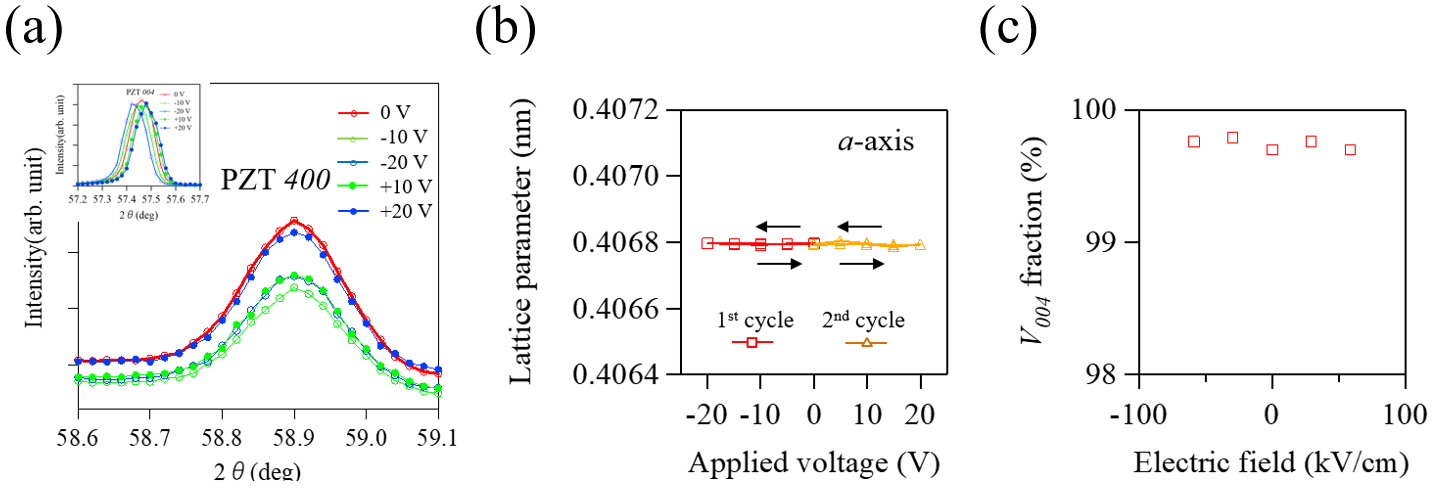


**Figure S3.** (a) In-situ XRD out-of-plane scans near the PZT *400* peak of the epitaxial PZT thin film under varying voltages. The insert shows the XRD patterns near the PZT *004* peak. (b) The variation in *a*-axis lattice parameter as a function of applied voltage. (c) The volume fraction (*V_004_*) of the *c*-domain as a function of electric field.

**4. Peak shift analysis in the polycrystalline PZT thin film**

As for the calculation of *c*-axis lattice parameter from d-spacing for a polycrystalline PZT thin film with a rhombohedral phase, we assumed that the rhombohedral angle was constant by setting the angle to 89.65˚ (Crystallography Open Database No.1538393). As described in the main text, the field-induced variation of the *c*-axis lattice parameter in the polycrystalline PZT thin film presents a butterfly curve due to inverse polarization. After the 2^nd^ cycle (see Fig. 2(b)), we applied negative voltages (forward bias) of -10 V, -20 V and -30 V for the 3^rd^ cycle, and then positive voltages (reverse bias) of +15 V, +20 V and +30 V for the 4^th^ cycle. Resulting in-situ XRD patterns for the 3^rd^ and 4^th^ cycles are shown in Fig. S4(a) and (b). We confirm that the variation in lattice parameter (Fig. S4(c)) exhibits a behavior similar to that obtained in the 1^st^ and 2^nd^ cycles, as shown in Fig. 2(b).


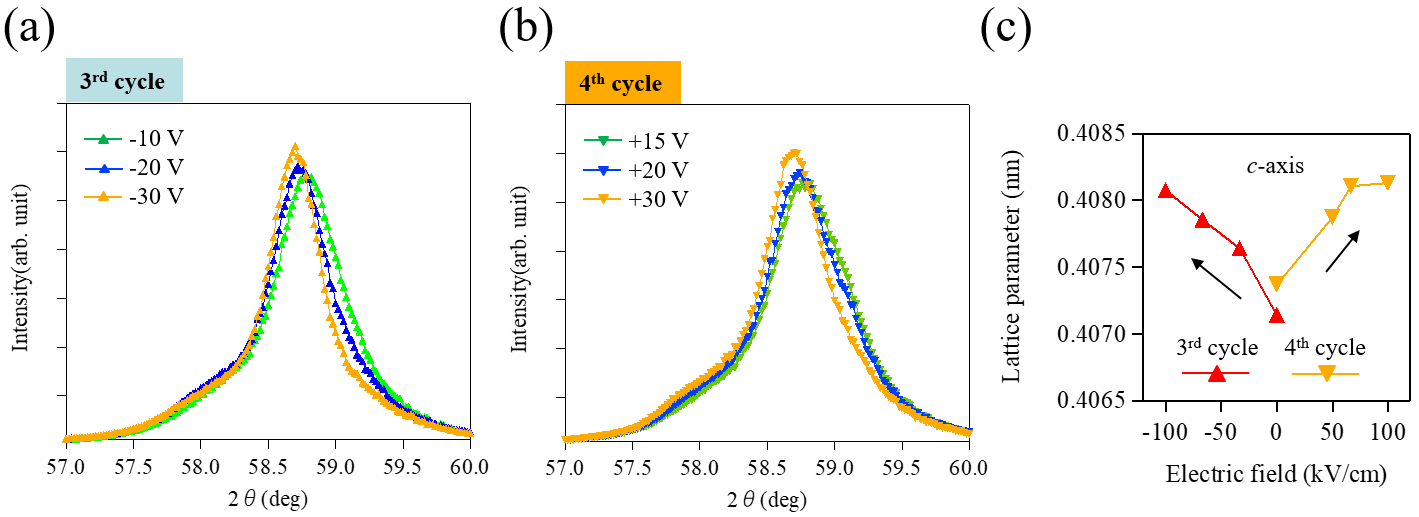


**Figure S4.** (a) XRD profiles of the polycrystalline PZT thin film for the 3^rd^ cycle using forward electric fields. (b) XRD profiles of the polycrystalline PZT thin film for the 4^th^ cycle using reverse electric fields. (c) The variation in lattice parameter of the polycrystalline PZT thin film for the 3^rd^ and 4^th^ cycles.
